# GATA3 is downregulated in HCC and accelerates HCC aggressiveness by transcriptionally inhibiting slug expression

**DOI:** 10.3892/ol.2021.12836

**Published:** 2021-06-02

**Authors:** Zhuoliang Zhang, Xingliang Fang, Guilin Xie, Jinlong Zhu

Oncol Lett 21: Article no. 231, 2021; DOI: 10.3892/ol.2021.12492

Following the publication of the above article, the authors have realized that, in the Declarations section on p. 10, they presented an incorrect approval number from the Ethics Committee in question; the statement here should have read as follows: “The present study was approved by the Ethics Committee of the Affiliated Hospital of Shaoxing University (approval no. **2021003**). All patients provided written informed consent.”

The authors regret their oversight in providing the incorrect approval number in the *Ethics approval and consent to participate* section of the paper. They thank the Editor of *Oncology Letters* for allowing them the opportunity to publish this corrigendum, and apologize to the readership of the Journal and to the Ethics Committee of the Affiliated Hospital of Shaoxing University for any inconvenience caused.

